# Mineral Waters of Texas

**Published:** 1890-06

**Authors:** Edgar Everhart

**Affiliations:** Chemist, University of Texas


For Daniel's Texas Medical Journal.

### THE MINERAL WATERS OF TEXAS.

BY EDGAR EVERHART, PH. D., CHEMIST, UNIVERSITY OF TEXAS.

THE MORE Texas is known, the more impressed is one with the immense number and variety of its natural products. Its iron fields are as fine and large as any in the world; in fact, the analysis of some of the ores made in the laboratory here has shown that they are superior to any yet known. The kaolin deposits in the State are perhaps finer than those of any other country. Many other minerals, too numerous to mention, are found in abundance and great purity within the confines of

Texas. Even gold and silver exist in large quantities, in several localities.

However interesting these facts may be to the general public, they do not concern the medical world as much as do the various mineral waters that occur in almost every portion of the State. A number of such waters have come to the University for analysis, and it is the purpose of this article to give the results of these analyses more particularly. It is a fact worthy of note that many of these mineral springs occur in those parts of the State that are most desirable as health resorts, the climate being good and the surroundings free from malaria. It is generally conceded that invalids are often as much benefitted by a change of climate, diet, etc., as they are by the medicinal virtues of the water. This being the case, it is a matter of considerable importance that the springs should be situated in a healthy locality.

Some of the mineral waters of the State have had a local reputation, at least, for a number of years, but so far as I have been able to find out, very few analyses have been made, and those incomplete. For example, the different waters of the more than locally-known Sour Lake, as reported in the Transactions, Texas State Medical Association, for 1884, are not only incomplete, but also inaccurately analyzed. Of the Wootan Wells water, no complete analysis has been seen by the writer; it is said, howeAer, to be an excellent chalybeate water. Of the other waters mentioned in the above report, the following analyses are given:

MINERAL WELLS, PALO PINTO COUNTY, TEXAS.

I make alterations in this report, so that it will conform to the ordinary practice of chemists. Thus, sodium sulphate is never reported as the hydrated salt, as given on page 210 of the Transactions for 1884, but always as the anhydrous. These corrections will lower the apparent solid constituents. One gallon of the water contains:

| | | |
|---|---|---|
| Sodium sulphate | 66.17 | grains |
| Magnesium sulphate | 9.19 | " |
| Calcium sulphate | 5.18 | " |
| Calcium carbonate | 2.08 | " |
| Magnesium carbonate | 4.66 | " |

| | | |
|---|---|---|
| Sodium chloride | 23.98 | grains |
| Calcium chloride | 1.28 | " |
| Alumina | 1.54 | " |
| Silica | 1.18 | " |
| Organic matter, volatile and loss | 9.80 | " |
| Trace of iron, iodine, ammonia, suspended matter, clay, etc | 11.58 | " |

ANALYSIS OF THE WEAVER WELL, SULPHUR SPRINGS, TEXAS

One gallon of the water contains:

| | | |
|---|---|---|
| Sulphate of ferric oxide | 33.42 | grains |
| Sulphate of alumina | 23.24 | " |
| Sulphate of magnesia | 24.34 | " |
| Sulphate of lime | 45.68 | " |
| Sulphate of soda | 1.34 | " |
| Sulphate of potash | 0.85 | " |
| Chloride of sodium | 1.36 | " |
| Carbonate of lime | 4.29 | " |
| Phosphate of lime | 0.63 | " |
| Silicic acid | 1.42 | " |
| Free sulphuric acid | 1.21 | " |
| Organic matter | 2.47 | " |

ANALYSIS OF WELL NO. 1, BRYAN, TEXAS.

One gallon of the water contains:

| | | |
|---|---|---|
| Chloride of sodium | 6.24 | grains |
| Sulphate of alumina | 1.49 | " |
| Sulphate of iron, alumina and manganese | 54.76 | " |
| Sulphate of lime | 44.99 | " |
| Sulphate of magnesia | 28.06 | " |
| Sulphate of zinc | trace. | |
| Silica | 6.45 | " |

ANALYSIS OF WELL NO. 2, BRYAN, TEXAS.

One gallon of the water contains:

| | | |
|---|---|---|
| Calcium sulphate | 45.66 | grains |
| Sodium chloride | 4.10 | " |
| Strontium carbonate | 0.27 | " |
| Iron in sulphate and carbonate | 10.59 | " |

| | |
|---|---|
| Silica | 7.51 grains |
| Manganese sulphate | 0.33 " |
| Sulphate of potassa | small quantity |
| Carbonic acid | abundant |

Of these waters, the analyses of which are given above, the first belongs to the class of saline sulphatic, the others are chalybeate.

The following list of watering places is taken from the Transactions, Texas State Medical Association for 1884, and, together with those to be mentioned below, a1e all that are known in our State at the present time. Whether analyses of these waters have been made or not, is uncertain. The importance of having such analyses made in unquestionable. The list is as follows:

Sour Lake, Harden county; Wootan Wells, Kendrick Wells, Fisher's Wells, Robertson county; Burdett's Wells, near Luling; McQueen's Wells, near Bryan; Mineral Wells, Palo Pinto county; Weaver's Wells, Pate's Wells, Sulphur Springs; Lampasas Springs, Lampasas; Thorp Springs, Hood county; Hugh's Springs, Cass county, Dalby Springs, Bowie connty.

Many of these waters may be very valuable, but our present ignorance of their constitution and properties renders it impossible even to classify them. It is to be hoped that the owners of these waters will take occasion to let the medical world know more particulars about them.

Almost all the different varieties of mineral waters are found iu the State. Many of them are of unusually fine quality, and are comparable with the most celebrated springs of this country and of Europe. The use of mineral waters is becoming more and more general throughout the civilized world. As remedial agents, they sometimes reach complaints and diseases that the physician, with all his skill, is unable to cope with. It would be out of place for a chemist to attempt to explain the therapeutic action of mineral water, and therefore I intend only to give the analysis of those products of our State, and to indicate the usual diseases for which like waters are elsewhere prescribed.

Probably the most common of the mineral waters in Texas are the chalybeate. Whether iron taken into the system by the drinking a water containing it, is absorbed, or whether it is entirely excreted, is still a mooted point among physicians. There is no question, however, about the good effects often produced by the use of these chalybeate waters, whether that use be internal or external. The iron salts are very frequently accompanied by other constituents that materially aid their action. The presence of sodium chloride, or sulphate, or carbonate, or free carbonic acid for example, are valuable adjuncts to such waters. Many female complaints, anæmia from almost any cause, diseases of the skin, ulcers, etc., are treated successfully by such waters. Remarkable cures by the chalybeate waters of Texas are on record. Closely allied to these are the alum waters. They are styptic and astringent. They are particularly useful for external application, and are prescribed for scrofula, anæmia, chronic diarrhœs, and various skin diseases. Good examples of this kind of water are found in several parts of the State, especially around Franklin, Robertson county. Below are given several analyses made in the laboratory of the University.

Analysis of chalybeate water No. 1 from Franklin, Texas. One gallon of the water contains:

| | | |
|---|---|---|
| Sulphate of lime | 20.965 | grains |
| Sulphate of iron | 75.952 | " |
| Sulphate of alumina | 13.063 | " |
| Sulphate of soda | 7.109 | " |
| Sulphate of potash | 0.752 | " |
| Sulphate of ammonia | trace | |
| Chloride of sodium | 9.715 | " |
| Silica and insoluble matter | 2.041 | " |
| Total grains per gallon | 131.825 | grains |

Reaction of the water slightly acid.

Analysis of chalybeate water No. 2, from Franklin, Texas. One gallon of the water contains:

| | | |
|---|---|---|
| Sulphate of lime | 10.42 | grains |
| Sulphate of iron | 8.61 | " |
| Sulphate of magnesia | 5.42 | " |
| Sulphate of soda | 2.64 | " |

| | | |
|---|---|---|
| Sulphate of potash | 0.11 | grains |
| Chloride of sodium | 3.GG | H |
| Silica and insoluble matter | 1.33 | " |
| Alumina and manganese | traces | |

Analysis of chalybeate water No. 3, from Franklin, Texas. One gallon of the water contains:

| | | |
|---|---|---|
| Sulphate of lime | 37.095 | " |
| Sulphate of iron | 144.903 | " |
| Sulphate of alumina | 4.998 | " |
| Sulphate of soda | 14.143 | " |
| Sulphate of potash | 2.560 | " |
| Chloride of sodium | 9.035 | " |
| Silica and insoluble matter | 2.244 | " |
| Total grains per gallon | 235.015 | grains |

An examination of these three waters will show that while No. 2 contains a moderate amount of the sulphate of iron, No. 1 contains a large quantity, and No. 3 so much as is rarely met with anywhere. No. 2 can be used for drinking, but the other two, when undiluted, are only good for bathing.

Good examples of alum waters are met with at Denison and also at Franklin.

Analysis of alum water from Denison, Texas. One gallon of the water contains:

| | | |
|---|---|---|
| Sulphate of alumina | 26.850 | grains |
| Sulphate of lime | 30.652 | " |
| Sulphate of iron | 0.239 | " |
| Sulphate of magnesia | 3.312 | " |
| Sulphate of potash | 0.286 | " |
| Chloride of sodium | 4.222 | " |
| Chloride of magnesium | 0.140 | " |
| Silica and insoluble matter | 2.735 | " |
| Total grains per gallon | 68.436 | grains |

Analysis of an alum water from Franklin, Texas. One gallon of the alum water contains:

| | | |
|---|---|---|
| Sulphate of alumina | 30.005 | " |
| Sulphate of lime | 6.217 | " |
| Sulphate of iron | 5 651 | " |
| Sulphate of magnesia | 2.624 | " |
| Sulphate of potash | 0.548 | " |
| Sulphate of soda | 0.898 | " |
| Silica and insoluble matter | 2.887 | " |
| Total grains per gallon | 52.772 | " |

A class of mineral waters has been recently discovered near Georgetown that are particularly valuable, and outside of Bohemia and Hungary are rarely met with. These waters are known as the strongly sulphated alkaline. They contain large amounts of Epsom's and Glauber's salts, and in the quantities of these two constituents, stand between the celebrated waters of Carlsbad and the Hunyadi Janos, the latter being more strongly purgative, and the former less than the Georgetown. If the water of these wells were evaporated, there would be obtained solid salts equal, if not superior, to the well-known Crab Orchard salts.

There are several of these wells near Georgetown, all of them having about the same constitution. One of the best representa tive is the Page well, the water of which was examined a year or two ago.

Analysis of the Page well, Georgetown, Texas. One gallon of water contains:

| | | |
|---|---|---|
| Chloride of sodium | 58.720 | grains |
| Bicarbonate of soda | 1.225 | " |
| Carbonate of lime | 23.414 | " |
| Carbonate of iron | 0.811 | " |
| Sulphate of alumina and potash | 8.363 | " |
| Sulphate of potash | 3.531 | " |
| Sulphate of soda | 201.197 | " |
| Sulphate of magnisia | 62.925 | -' |
| Sulphate of lime | 27.543 | " |
| Silica and insoluble matter | 0.991 | " |
| Total grains per gallon | 381.125 | " |

Free carbonic acid gas, 39.6 cubic inch per gallon.

Waters like the above have been successfully used for obesity, catarrh of the stomach, affections of the biliary organs, gravel, calculus, etc. They are usually strongly diuretic.

Some of the most valuable mineral waters of the State are found at Lampasas. They are interesting not only from a medical, but also from a physical standpoint. A number of sulphur springs exist at Lampasas, and they may be divided into two groups situated about one mile apart. Curiously enough, although the flow of water in all the springs remains constant, yet the presence of sulphur alternates between the two groups. When the upper springs are strong in sulphur, the lower ones are practically free from that ingredient, and when the lower are snlphuretted the upper ones are not. No plausible explanation of this phenomenon has been suggested. The Lampasas sulphur waters are equal to the best known. Waters of this class are given in dartrous skin diseases, diseases of the bladder, jaundice, dyspepsia, scrofula, syphilis, etc.

ANALYSIS OF THE HANNA SPRING, LAMPASAS, TEXAS.

One gallon of the water contains:

| | | |
|---|---|---|
| Hyposulphite of soda | 0.775 | grains |
| Hydrosulphate of sodium | 0.111 | " |
| Chloride of sodium | 555.869 | " |
| Chloride of potassium | 10.287 | " |
| Chloride of magnesium | 50.049 | " |
| Chloride of calcium | 48.905 | " |
| Carbonate of lime | 34.465 | " |
| Sulphate of lime | 0.951 | " |
| Bromide of magnesium | trace | |
| Alumina | 2.286 | " |
| Silica and insoluble matter | 1.673 | " |
| Total grains per gallon | 705.371 | |

Free sulphuretted hydrogen gas, 5.82 cu. in. per gallon.

Free carbonic acid gas, 11.80 cu. in. per gallon.

Reaction, faintly acid.
Temperature of the water, 70 degrees F.
Specific gravity, 1.0074.

ANALYSIS OF THE COOPER SPRING, LAMPASAS, TEXAS.

One gallon of the water contains:

| | | |
|---|---|---|
| Hyposulphite of soda | 0.227 | grains |
| Hydrosulphate of sodium | 0.827 | " |
| Chloride of sodium | 346.974 | " |
| Chloride of potassium | 3.586 | " |
| Chloride of magnesium | 21.857 | " |
| Chloride of calcium | 48.147 | " |
| Sulphate of lime | 4.461 | " |
| Carbonate of lime | 11.372 | " |
| Bromide of magnesium | trace | |
| Alumina | 3.540 | " |
| Silica and insoluble matter | 1.673 | " |
| Total grains per gallon | 441.555 | |

Reaction, faintly acid.
Temperature of the water, 70 degrees F.
Specific gravity, 1.007.
The other springs have water of like composition.

Near the town of Sunset, Texas, there is a purely saline water, which resembles that of Kreuznach, Germany. Waters of this kind are used more for bathing than for drinking. They are prescribed for scrofula, anaemia, and skin diseases.

ANALYSIS OF SALINE SPRING NEAR SUNSET, TEXAS.

One gallon of the water contains:

| | | |
|---|---|---|
| Chloride of sodium | 87.01 | grains |
| Chloride of magnesium | 27.88 | " |
| Chloride of calcium | 116.26 | " |
| Sulphate of lime | 48.46 | " |
| Carbonate of lime | 81.64 | " |
| Total grains per gallon | 361.25 | |

In the sinking of an artesian well on the outskirts of Austin, a valuable mineral water was obtained. The water resembles, in its properties, the well known Vichy. It is also very similar to the Saratoga, except that the latter contains much more common salt and less sulphate of soda. There is a faint trace of sulphuretted hydrogen in the water which imparts to it a slight and not unpleasant taste. The water is warm and belongs to a highly prized class. It may be used in gout, lithiasis, affections of the liver, catarrh, and obstructions of the gall duct, in dyspepsia, chronic catarrh of the stomach, and diarrhœa, in obesity and in diabetes.

ANALYSIS OF THE GROOM MINERAL WATER, AUSTIN, TEXAS.

One gallon of the water contains:

| | | |
|---|---|---|
| Bicarbonate of soda | 19.07 | grains |
| Bicarbonate of lithia | trace | " |
| Carbonate of lime | 14.58 | " |
| Carbonate of magnesia | 7.25 | " |
| Sulphate of potash | 1.67 | " |
| Sulphate of aoda | 51.01 | " |
| Sulphate of lime | 2.15 | " |
| Chloride of sodium | 17.35 | " |
| Bromide of sodium | 0.34 | " |
| Iodide of sodium | 0.03 | " |
| Phosphate of soda | 0.06 | " |
| Sulphale of strontia | 0.59 | " |
| Sulphate of baryta | trace | |
| Alumina | 0.04 | " |
| Oxide of iron | 0.02 | " |
| Silica | 1.16 | " |
| Total grains per gallon | 118.32 | |

Free carbonic acid gas, 79. 12 cu. in. per gallon.

Free sulphuretted hydrogen gas, 0.15 cu. in. per gallon.

Temperature of the water, 88 degrees F.

An examination of the analysis given above will show that Texas is rich not only in the number of its mineral waters, but also in their quality.

